# MK4MDD: A Multi-Level Knowledge Base and Analysis Platform for Major Depressive Disorder

**DOI:** 10.1371/journal.pone.0046335

**Published:** 2012-10-05

**Authors:** Liyuan Guo, Weina Zhang, Suhua Chang, Liuyan Zhang, Jurg Ott, Jing Wang

**Affiliations:** 1 Key Laboratory of Mental Health, Institute of Psychology, Chinese Academy of Sciences, Beijing, China; 2 Graduate University of Chinese Academy of Sciences, Beijing, China; University of Illinois at Chicago, United States of America

## Abstract

**Background:**

Major depressive disorder (MDD) is a complex neuropsychiatric syndrome with high heterogeneity. There are different levels of biological components that underlie MDD and interact with each other. To uncover the disease mechanism, large numbers of studies at different levels have been conducted. There is a growing need to integrate data from multiple levels of research into a database to provide a systematic review of current research results. The cross level integration will also help bridge gaps of different research levels for further understanding on MDD. So far, there has been no such effort for MDD.

**Descriptions:**

We offer researchers a Multi-level Knowledge base for MDD (MK4MDD) to study the interesting interplay of components in the pathophysiological cascade of MDD from genetic variations to diagnostic syndrome. MK4MDD contains 2,341 components and 5,206 relationships between components based on reported experimental results obtained by diligent literature reading with manual curation. All components were well classified with careful curation and supplementary annotation. The powerful search and visualization tools make all data in MK4MDD form a cross-linked network to be applied to a broad range of both basic and applied research.

**Conclusions:**

MK4MDD aims to provide researchers with a central knowledge base and analysis platform for MDD etiological and pathophysiological mechanisms research. MK4MDD is freely available at http://mdd.psych.ac.cn.

## Introduction

Major depressive disorder (MDD) is a common neuropsychiatric syndrome with a life time prevalence of ∼17% of the population worldwide [1]. It has been predicted to become the second leading cause of disability worldwide by 2020 [2] and will cause a high global burden. As a complex disease, MDD is influenced by both genetic and environmental factors, with a heritability of approximately 37% [3]. Meanwhile, it presents symptomatic heterogeneity and involves changes in multiple systems [4], [5]. The high complexity of cause and mechanism of MDD leads to difficulties in its early detection, treatment and prognosis.

In the last decade, supported by theoretical and technological developments, substantial advances have been made in MDD research. For example, several hypotheses on MDD pathophysiological mechanisms have been supported by evidence from molecular neurobiology [5], [6]; large numbers of genetic studies, especially several genome-wide association studies, were carried out to identify MDD risk loci [7], [8], [9], [10], [11], [12], [13]; many studies emerged to discuss gene-environment interplay in MDD [14], [15], [16]; a series of new methods and research models have been applied in psychiatric research, such as next-generation sequencing technique [17], neuroimaging [18], and animal models [19]. Despite these advances, there are still various challenges. Towards the goal of uncovering mechanism of psychiatric disease, researchers from multiple disciplines tend to, on the one hand, narrow research targets to examine specific components of disease processes, thereby potentially improving the focus of investigation [20]; on the other hand, there is an increasing trend to integrate multiple levels of research data and technologies towards a systematic understanding of disease psychopathology [20], [21]. For example, there have been studies to examine the influence of genetic variation on gene expression and brain structure and function for psychiatric disorders [21], [22], [23], [24]. All these efforts accelerated the accumulation of related data and advanced our knowledge on MDD.

However, results from different fields of research are scattered in numerous publications, and there is a lack of a systematic review and collection of the currently available data and knowledge. Meanwhile, research at different levels remains fragmented, which hinders interdisciplinary research to articulate multiple levels of analysis. To solve these problems, one of the biggest challenges is the lack of tools to manage the complexity of data rapidly being accumulated across widely disparate methods, models and data types [25]. So a database to integrate data of different research levels will greatly facilitate MDD studies and will provide unique insights into how the dynamic interplay between different levels of data shapes individual risk for psychopathology. There have been some databases for psychiatric disorders currently available, including AutDB for autism [26], SZGene [27] and SZGR [28] for schizophrenia, and ADHDgene for attention deficit/hyperactivity disorder [29]. Although there are some disease-related databases such as SLEP [30] and HuGE Navigator [31] which collected genetic information for a set of disorders including MDD, they are not MDD specific. A recent publication reported a prioritized gene list (DEPgenes) for MDD by gene prioritization analysis [32], but the result is from prediction instead of literature-origin and the data is not available by database or download. More importantly, all the currently available databases focus on the genetic basis of psychiatric disorders. MDD is a complex neuropsychiatric syndrome with great heterogeneity. Besides the genetic basis, it is of critical importance to focus on multiple levels of biological characteristics to establish a comprehensive multi-level knowledge base for MDD.

The database of Multi-level Knowledge base for MDD (MK4MDD) has thus been developed as an innovative informatics tool to integrate different levels of data in published experimental studies of MDD. MK4MDD provides researchers a knowledge network, which contains integrated data and the interplay between data of different levels. It is also an analysis platform in which online customized analysis with vivid visualization could be done. By developing MK4MDD, we aim to facilitate a broad range of works on both MDD basic research and practical applications, and ultimately to facilitate an understanding of MDD mechanism and development of effective means for disease diagnosis, treatment and prognosis. Our innovative database schema could be a framework to be applied to the study of other complex psychiatric diseases.

## Materials and Methods

### Literature Search and Data Extraction

MK4MDD aims to provide multi-level data that cover the pathophysiological cascade of MDD from genetic variations to diagnostic syndrome. The pathophysiological cascade was classified as different levels of gene, protein, cellular system/signaling pathway, neural system, cognition, and symptom by taking reference of the paper published by Cannon TD *et al* [33]. A search formula *(“major depression”[Title/Abstract] OR “MDD”[Title/Abstract] OR “unipolar depression”[Title/Abstract] OR “unipolar depressive disorder”[Title/Abstract] OR “major depressive disorder”[Title/Abstract]) AND (“XXX”[Title/Abstract] OR “XXX”[Title/Abstract] OR … OR “XXX”[Title/Abstract])* was designed to search publications from PubMed. The five aliases of MDD were gotten from Wikipedia and Medical Subheadings (MeSH) terms of MDD; other search keywords (represented as XXX in the formula) were selected by taking reference of published papers [5], [6], [26], [27], [28], [29], [33], [34], [35], [36], [37], [38], [39], [40], [41] which were elaborated in supplementary file. The searches resulted in 5,886 publications from the year 2001 through March 1st, 2012. By manually screening of these publications, MDD-related genetic studies, epigenetic studies, functional studies including animal models, imaging studies and psychological studies focusing on the etiological and pathophysiological mechanisms of MDD were retained. Studies about reliability of psychometric scales, epidemiology, and efficacy of anti-depressed drugs or treatment were not included. Finally, after filtering, there are 1,462 articles included in MK4MDD.

The abstract of each eligible publication was read carefully and data was extracted strictly sticking to the original publication. In MK4MDD, specific data of a certain research level is defined as a ‘component’ of disease process, and the association between two components of either the same research level or different levels is defined as a ‘relationship’. For example, one study reported that ‘significantly smaller hippocampal volumes were observed for patients and for controls carrying the Met-BDNF allele compared with subjects homozygous for the Val-BDNF allele (*P* = 0.006).’ [42]. Based on this result, two components can be extracted including *BDNF* gene and hippocampus, and one relationship between these two components can be established as described in the original paper. For relationships, detailed information like statistical value (*e.g. P*-value, odds ratio, confidence interval), relation description were extracted. To facilitate an understanding of relationships, other detailed information of the study including background, sample information, method, result and conclusion, were also provided. Moreover, environmental events reported in publications from the search results of keywords in the table in Supplementary File S1, which might be putative risk factors for MDD, were also included into the database.

### Data Integration and Analysis

The data from literature were classified into seven research levels according to pathophysiological cascade of MDD and data in different levels were integrated by relationships between components. The seven levels are: (1) genetic/epigenetic locus, (2) protein and other molecule, (3) cell and molecular pathway, (4) neural system, (5) cognition and behavior, (6) symptoms and signs, and (7) environment. The seven research levels were further classified into 14 data types. Detailed descriptions of data types and research levels are shown in Table 1. All components in MK4MDD are classified into appropriate data type in appropriate level. For instance, the two components mentioned in the above example, *BDNF* gene and hippocampus, are classified into ‘gene’ type in ‘genetic/epigenetic locus’ level, and ‘brain morphology and function’ type in ‘neural system’ level, respectively.

**Table 1:** Descriptions of data types and research levels.

Data level/type	Type description
*Genetic/epigenetic locus*	
SNP	Single nucleotide polymorphisms
Gene	Protein coding genes
Region	Genomic regions
Epigenetic site	Epigenetic modification sites in chromatin
*Protein and other molecule*	
Protein	MDD related proteins
Molecule	Small molecules (such as transmitters)
*Cell and molecular pathway*	
Molecular pathway	Intracellular or intercellular molecular pathway that have been recorded in GO [42], Biocarta (http://www.biocarta.com/genes/index.asp), or KEGG [43].
Cell	Cell type such as pyramidal neuron, lymphocytes.
*Neural system*	
Neurobiological system	Electroneurographic signals and comprehensive systems that usually contain multiple molecules, molecular pathways and cells (such as transmitter system).
Brain morphology and function	MDD related structural and functional brain changes.
*Cognition and behavior*	Cognitive impairments and cognitive characteristics of MDD patients, as well as depression/anxiety-like behaviors from animal models
*Symptoms and signs*	
Symptoms	Diagnostic symptoms for MDD in DSM-IV (except cognitive impairments).
Signs	Clinical signs, such as blood pressure or heart rate.
*Environment*	Environmental events that are putative risk factors for MDD

**Figure 1:**
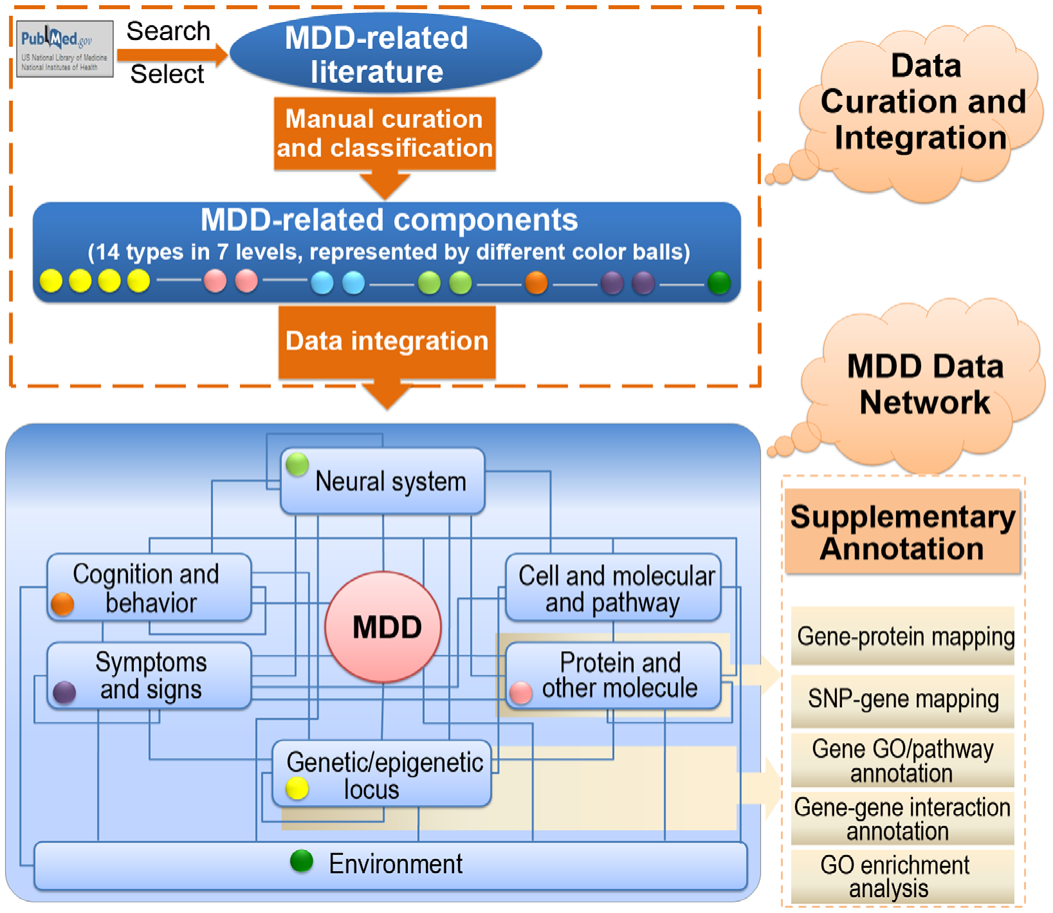
The process of data integration and analysis and the data schema in MK4MDD. SNP: single nucleotide polymorphism, GO: gene ontology.

Careful curation and annotation were made on each component in a manual or semi-manual manner by reading reviews, textbooks or searching databases. For example, the name of component was standardized according to common databases or knowledge, such as finding approved symbols for genes in HGNC [43], and approved names for proteins in UniProt [44]. Detailed descriptions were provided for some types of data, such as diagrammatic presentation for neural system components and morphological and functional annotation for brain [45], [46], [47]. For components of SNPs, genes, proteins and molecular pathways, supplementary annotations were made for a deeper interpretation of current data, as some important annotations were not provided directly in original publications. The supplementary annotations include functional annotation for SNPs (non-synonymous coding SNPs, or SNPs leading to gain or loss of stop codon) using dbSNP [48] and Ensembl [49], mapping SNPs to genes according to their chromosomal locations, annotating genes by using gene ontology (GO) [50] and pathways from KEGG [51], BioCarta (http://www.biocarta.com/genes/index.asp) and Reactome [52], GO enrichment analysis by DAVID [53], [54] for genes, mapping genes to proteins by using the UniProt [44] database, and identifying interactions between genes by using the HPRD [55] database. On the other hand, hot components (defined as components with at least *N* studies, in which, the threshold *N* is different for different data type) were analyzed for each data type to provide reliable candidates for further research. These supplementary analyses enriched the content of the database to provide new clues for understanding the genetic basis and molecular mechanism of MDD. The process of data integration and analysis, as well as data schema to show the different levels of data and relationships among them are shown in Figure 1.

## Results and Application

### Data Content and Data Access

The data set of MK4MDD contains 2,341 components and 5,206 relationships, which are all based on reported experimental results from the literature. Statistics for each type of data are shown in Table 2. Supplementary annotations for components in the ‘genetic/epigenetic locus’ level and the ‘protein and other molecule’ level were included in MK4MDD. All data of MK4MDD are stored and managed by a MySQL relational database. To access the data, MK4MDD provides researchers a user friendly interface with powerful search and visualization tools developed by using Java/JSP running on an Apache Tomcat web server. There are several search options to facilitate access to the MDD data network. The ‘Multi-level Search’ allows users to search published reports of a given component of interest in all the seven research levels, so that researchers may acquire a systematic review of the current research status of the component. A blank input will result in a list of all MDD related components classified by research levels for a panoramic overview of MDD study. Among the seven research levels, in consideration of the genetic complexity of MDD, a special module of ‘Genetic/Epigenetic Locus Search’ is designed to facilitate a thorough investigation on genetic or epigenetic data. Particularly, to well demonstrate data connections, ‘Cross Data Search’ is implemented to search relationships between different levels of components. For example, to select the level of component A as ‘Gene’ and level of component B as ‘Brain Morphology and Function’, as well as to select ‘Positive’ in the field of ‘Relation Type’ and ‘Patients study’ and ‘Patients and normal controls study’ in the field of ‘Study Type’, will acquire 95 positive relationships between genes and components of brain morphology and function from studies of patients or patients and normal controls.

**Table 2:** MK4MDD data content and statistics as of 1 March 2012.

Data type	Number of components
SNP	260
Gene	675
Region	32
Epigenetic site	450
Protein	302
Molecule	82
Molecular pathway	53
Cell	24
Neurobiological system	39
Brain morphology and function	326
Cognition and behavior	42
Symptoms	21
Signs	19
Environment	16
**Total number of components**	2,341
**Total number of relationships between two components**	5,206
**Number of articles**	1,462

MK4MDD provides a detailed report for each component, which mainly includes the following information:

- ‘*Basic information*’. This part includes basic information for components. For example, in ‘Gene Report’, it includes gene symbol, name, alias, location, position, external links and number of articles that reported the relationships between gene and MDD, and between gene and other components.
- ‘*Relationship between component and MDD*’. This part presents a detailed description of the relationship between components and MDD, including a list of references which reported the relationship, statistical result and relation description in each reference followed the original publication. Positive and negative relationships are presented separately. The reference is linkable and detail information about specific study including sample size, method is presented in the linked study report.
- ‘*Relationship between component and other components at different levels*’. This is to demonstrate how components are linked with each other. Components at different levels are denoted by balls of different colors. Highlighted balls indicate there are components at that level that have reported relationship with the component under study. Users may click on a highlighted ball to view details. In this part, positive results and negative results are shown separately too.
- ‘*Data network of component*’. A graphical data network, centered by the component under study and constituted of its related components which have positive results described in (b) and (c), will be generated automatically by using Cytoscape Web [56] to show their relationships. Together with zoom in/out function, the dynamic network allows researchers to get a vivid view of data and data connections. Users may view the detailed report for each component (*i.e.* node in network) simply by clicking on the colored balls.
- ‘*Supplementary annotation*’. This part of information was acquired by bioinformatic analysis, and is only available for components of SNPs, genes, proteins, and pathways. For example, in ‘Gene Report’, the supplementary annotation includes gene related SNPs and proteins, GO annotation for gene functions, gene related pathways, and interactions between genes.

To help users acquire customized data network, MK4MDD provides an unique module named ‘My Relationship Set’, which allows users to add selected positive relationships to establish users’ own relationship set by clicking on the ‘ADD’ button from either ‘Component Report’ or search results of ‘Cross Data Search’. Users can not only add literature-origin relationships into the ‘My Relationship Set’, but also relationships from supplementary annotation. On the ‘My Relationship Set’ page, users can do further editing on the selected relationships to generate a graphical data network, where relationships of literature-origin or from supplementary annotation are differentiated by using solid and dot lines, respectively. Users can drag or click on the nodes/edges for interactive operations and analysis. Users may also store their relationship set by using the download function and upload the set for further analysis when needed.

## Application

MK4MDD is an innovative informatics tool to facilitate studies about etiological and pathophysiological mechanisms of MDD. By managing and integrating the complex data and data connections, researchers may start from a single component of interest to acquire a knowledge network across different research levels. MK4MDD will have a broad range of applications, both in basic and applied research, to advance our knowledge on MDD mechanism and for development of effect means for early detection and treatment of the disease.

**Figure 2:**
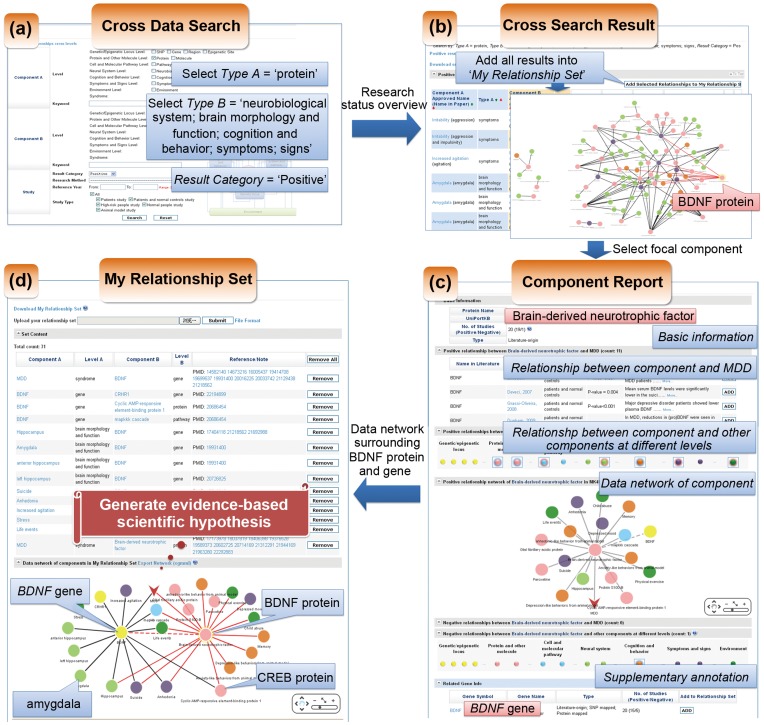
An example of the application of MK4MDD in deriving evidence-based new hypothesis for MDD research.

For basic research, MK4MDD provided a systematic review for MDD related components which will help to advance hypothesis-driven research. Here we use an example to demonstrate how MK4MDD will facilitate generating new questions. It is well-known that molecular mechanisms of MDD, especially what roles proteins play in MDD, are still unclear. To investigate this, it is necessary to get an overview of the current research status (*i.e.* reported data and data relationships between data) and then derive a new hypothesis. To achieve this, first, by using ‘Cross Data Search’, we searched for positive relationships between ‘Protein’ (Component A) and ‘Neural system’, ‘Cognition and Behavior’, ‘Symptom and Signs’ (Component B) (Figure 2 (a)). There are a total of 144 results. Second, we added all search results into a ‘My Relationship Set’ for visualization of components and relationships, there are total of 132 non-repeated relationships (Figure 2 (b)). Based on the graphical presentation, we found that brain-derived neurotrophic factor (BDNF) is one of the key focal nodes, so we selected BDNF for further study and deleted the previous 132 relationships from the broad range of searches to improve the focus of investigation. By ‘Protein Report’ for BDNF, we obtained 16 components related with BDNF protein, as well as the relationship between BDNF protein and *BDNF* gene from supplementary annotation (Figure 2 (c)). By ‘Gene Report’ for *BDNF* gene, we acquired 12 components related with *BDNF* gene. We added all 28 relationships surrounding both BDNF protein and *BDNF* gene into the ‘My Relationship Set’. Finally this iterative analytical process resulted in 29 relationships (including the relationship between BDNF protein and *BDNF* gene) in the ‘My Relationship Set’. From the graphical presentation of components and relationships (Figure 2 (d)), we found that BDNF protein is connected with protein CERB (cyclic AMP-responsive element-binding protein 1) and *BDNF* gene is connected with both protein CERB and brain region amygdala. Based on this information, we may propose several questions. For example, would BDNF contribute to MDD by regulating CREB in the brain region of amygdala? What cognitive impairments will be caused by molecular activation of the above mechanism? Do BDNF related symptoms (depression mood, anhedonia and suicide) appear based on this potential mechanism? New questions will help drive new findings to accelerate our knowledge on MDD mechanism.

Another example of application is on study of endophenotypes. As researchers tend to improve the focus of investigation by examining specific components of disease processes, the study of endophenotypes becomes more and more important in uncovering disease mechanism and developing effective method for disease diagnosis. The term ‘endophenotype’ is defined as an internal phenotype that fills the gap between available descriptors, the gene and the elusive disease process [57]. It should fulfill the following criteria: (1) associated with the illness in population, (2) heritable, (3) state-independent, (4) found in unaffected family members at a higher rate than in the general population, and (5) shown to co-segregate with the illness within families [58]. As MK4MDD collects different types of disease-related components in MDD patients, unaffected first-degree relatives and healthy controls, it will assist in identifying the basis of biological or clinical plausibility of a putative endophenotype. For example, through ‘Cross Data Search’, we found six components in neurobiological system associated with MDD by setting the ‘Study Type’ to ‘High-risk people study’. The results provided proofs on disease association, familial association and heritability for the six neurobiological components. Among the six components, we focused on an event-related component named P300. From the ‘Neurobiological Component Report’ for P300, we found a reference to support the state-independence of P300 [59], and the data network of P300 showed that P300 is associated with protein S100B and psychotic symptoms, which prove its biological and clinical plausibility. Based on all of the above evidence, we may choose P300 as a potential endophenotype for further study.

To facilitate the discovery of potential biomarkers is an important application of MK4MDD in applied research. The definition of a biomarker from the National Institutes of Health (NIH) is ‘a characteristic that is objectively measured and evaluated as an indicator of normal biologic processes, pathogenic processes, or pharmacologic responses to a therapeutic intervention’ [60]_ENREF_39. Generally a biomarker is anything that can be used as an indicator of a particular disease state [60]. In the past years, continuous efforts have been made in looking for suitable biomarkers as diagnostic or therapeutic targets [61], [62], [63]. To provide new clues on biomarkers, MK4MDD provides a list of potential biomarkers by analyzing the current research data. There are several principles applied to the analysis: 1) directly related to MDD and reflected differences between patients and normal controls; 2) measured from peripheral blood, cerebrospinal fluid or other clinic common samples, or recorded from neural imaging methods; and 3) shown in a verifiable way (such as protein concentration, SNP genotype and brain volume). For example, using magnetic resonance spectroscopy (MRS), a study observed significantly reduced glutamate levels in the patients’ left cingulum compared to healthy controls [64]_ENREF_53. The study shown glutamate is related to MDD and can be measured and evaluated by MRS, so it can be regarded as a potential biomarker. There are a total of 480 potential biomarkers at different research levels according to our analysis. Users may search potential biomarkers by specifying the name, or defining research levels or test methods. Detailed evidence for each potential biomarker is provided in a corresponding ‘Component Report’.

## Discussion and Future Development

MK4MDD contains highly integrated knowledge from PubMed with manual curation and supplementary annotation. It has three notable features: 1) MK4MDD is the first database specific for MDD and contains multi-level data. Different types of MDD related data were collected and further categorized into seven levels in the pathophysiological cascade of MDD from genetic variations to diagnostic syndrome, which allows researchers to acquire systematic knowledge for MDD from the database. 2) MK4MDD emphasizes on the interplay of different levels of data across widely disparate disciplines, methods and data types by collecting literature-reported relationships, so that all data in MK4MDD form a cross-linked data network. Researchers may start from a single type of data (*e.g.* gene or protein) to acquire a knowledge network from molecular level to mind level by navigating MK4MDD. 3) MK4MDD is not only a knowledge resource, but also an analysis platform for interdisciplinary research to accelerate the pace of new discovery in psychotic mechanism. The powerful search and visualization tools enable access to both data and data connections for identification of novel targets and questions. Through the module of ‘My Relationship Set’, researchers can easily target, evaluate, and prioritize MDD related components of interest for future research.

It is worth mentioning that all components and relationships included in MK4MDD are based on experimental results of MDD reported by original articles. Relationships inferred by review articles and studies for people with depressed mood or trait but not diagnosed patients were not included in MK4MDD. In the next few years, the number of MDD studies is expected to keep increasing especially with the development of new research strategies and new technologies. MK4MDD will be periodically updated to ensure a most up-to-date follow up of the research progress of MDD. Moreover, the current version of MK4MDD focuses on core pathophysiological components of MDD, so the social and environmental factors are not included in the current focuses. In future, MK4MDD will continue to expand its coverage to these fields. Meanwhile, we will extend the functionality of MK4MDD by adding new modules for endophenotype study and gene-environment interaction study. MK4MDD is dedicated to provide a powerful and useful MDD resource for researchers. However, it is really a challenge to cover all relevant terms of such a complex human disorder with broad clinical manifestations and multiple risk factors. We encourage researchers to propose new search terms or to upload articles by providing PMID via the specially-designed ‘Upload’ module on the website.

As the first multi-level knowledge base for complex psychiatric disease, MK4MDD demonstrates a novel research framework for interdisciplinary research, which could be applied to other psychiatry disorders, such as schizophrenia and bipolar disorder. We believe that with the support from bioinformatics tools and emergence of new data and knowledge, future investigations will accelerate the pace of new discoveries. We hope our continuous efforts will help to unveil the psychiatric mechanism of MDD and to contribute to global mental health.

## Supporting Information

Supplementary File S1 **Criteria for keywords selection and all keywords employed to search MDD related publications in PubMed for MK4MDD.**
(DOC)

## References

[1] Kessler RC, Berglund P, Demler O, Jin R, Koretz D, et al (2003) The epidemiology of major depressive disorder: results from the National Comorbidity Survey Replication (NCS-R). JAMA 289: 3095–3105. 12813115 10.1001/jama.289.23.3095

[2] Murray CJ, Lopez AD (1996) Evidence-based health policy–lessons from the Global Burden of Disease Study. Science 274: 740–743. 8966556 10.1126/science.274.5288.740

[3] Sullivan PF, Neale MC, Kendler KS (2000) Genetic epidemiology of major depression: review and meta-analysis. Am J Psychiatry 157: 1552–1562. 11007705 10.1176/appi.ajp.157.10.1552

[4] Nestler EJ, Barrot M, DiLeone RJ, Eisch AJ, Gold SJ, et al (2002) Neurobiology of depression. Neuron 34: 13–25. 11931738 10.1016/s0896-6273(02)00653-0

[5] Belmaker RH, Agam G (2008) Major depressive disorder. N Engl J Med 358: 55–68. 18172175 10.1056/NEJMra073096

[6] Krishnan V, Nestler EJ (2008) The molecular neurobiology of depression. Nature 455: 894–902. 18923511 10.1038/nature07455 PMC2721780

[7] Shyn SI, Shi J, Kraft JB, Potash JB, Knowles JA, et al (2011) Novel loci for major depression identified by genome-wide association study of Sequenced Treatment Alternatives to Relieve Depression and meta-analysis of three studies. Mol Psychiatry 16: 202–215. 20038947 10.1038/mp.2009.125 PMC2888856

[8] Shi J, Potash JB, Knowles JA, Weissman MM, Coryell W, et al (2011) Genome-wide association study of recurrent early-onset major depressive disorder. Mol Psychiatry 16: 193–201. 20125088 10.1038/mp.2009.124 PMC6486400

[9] Lewis CM, Ng MY, Butler AW, Cohen-Woods S, Uher R, et al (2010) Genome-wide association study of major recurrent depression in the U.K. population. Am J Psychiatry 167: 949–957. 20516156 10.1176/appi.ajp.2010.09091380

[10] Wray NR, Pergadia ML, Blackwood DH, Penninx BW, Gordon SD, et al. (2010) Genome-wide association study of major depressive disorder: new results, meta-analysis, and lessons learned. Mol Psychiatry. doi: 10.1038/mp.2010.109. 10.1038/mp.2010.109 PMC3252611 21042317

[11] Bosker FJ, Hartman CA, Nolte IM, Prins BP, Terpstra P, et al (2011) Poor replication of candidate genes for major depressive disorder using genome-wide association data. Mol Psychiatry 16: 516–532. 20351714 10.1038/mp.2010.38

[12] Rietschel M, Mattheisen M, Frank J, Treutlein J, Degenhardt F, et al (2010) Genome-wide association-, replication-, and neuroimaging study implicates HOMER1 in the etiology of major depression. Biol Psychiatry 68: 578–585. 20673876 10.1016/j.biopsych.2010.05.038

[13] Sullivan PF, de Geus EJ, Willemsen G, James MR, Smit JH, et al (2009) Genome-wide association for major depressive disorder: a possible role for the presynaptic protein piccolo. Mol Psychiatry 14: 359–375. 19065144 10.1038/mp.2008.125 PMC2717726

[14] Caspi A, Sugden K, Moffitt TE, Taylor A, Craig IW, et al (2003) Influence of life stress on depression: moderation by a polymorphism in the 5-HTT gene. Science 301: 386–389. 12869766 10.1126/science.1083968

[15] Brendgen M, Vitaro F, Boivin M, Girard A, Bukowski WM, et al (2009) Gene-environment interplay between peer rejection and depressive behavior in children. J Child Psychol Psychiatry 50: 1009–1017. 19486224 10.1111/j.1469-7610.2009.02052.x

[16] Middeldorp CM, Cath DC, Beem AL, Willemsen G, Boomsma DI (2008) Life events, anxious depression and personality: a prospective and genetic study. Psychol Med 38: 1557–1565. 18294422 10.1017/S0033291708002985

[17] Avramopoulos D (2010) Genetics of psychiatric disorders methods: molecular approaches. Psychiatr Clin North Am 33: 1–13. 20159337 10.1016/j.psc.2009.12.006 PMC2843402

[18] Savitz JB, Drevets WC (2009) Imaging phenotypes of major depressive disorder: genetic correlates. Neuroscience 164: 300–330. 19358877 10.1016/j.neuroscience.2009.03.082 PMC2760612

[19] Schmidt MV (2011) Animal models for depression and the mismatch hypothesis of disease. Psychoneuroendocrinology 36: 330–338. 20674180 10.1016/j.psyneuen.2010.07.001

[20] Andreasen NC (1997) Linking mind and brain in the study of mental illnesses: a project for a scientific psychopathology. Science 275: 1586–1593. 9054346 10.1126/science.275.5306.1586

[21] Caspi A, Moffitt TE (2006) Gene-environment interactions in psychiatry: joining forces with neuroscience. Nat Rev Neurosci 7: 583–590. 16791147 10.1038/nrn1925

[22] Glahn DC, Paus T, Thompson PM (2007) Imaging genomics: mapping the influence of genetics on brain structure and function. Hum Brain Mapp 28: 461–463. 17471577 10.1002/hbm.20416 PMC6871349

[23] Hyde LW, Bogdan R, Hariri AR (2011) Understanding risk for psychopathology through imaging gene-environment interactions. Trends Cogn Sci 15: 417–427. 21839667 10.1016/j.tics.2011.07.001 PMC3163727

[24] Kohli MA, Lucae S, Saemann PG, Schmidt MV, Demirkan A, et al (2011) The neuronal transporter gene SLC6A15 confers risk to major depression. Neuron 70: 252–265. 21521612 10.1016/j.neuron.2011.04.005 PMC3112053

[25] Sabb FW, Bearden CE, Glahn DC, Parker DS, Freimer N, et al (2008) A collaborative knowledge base for cognitive phenomics. Mol Psychiatry 13: 350–360. 18180765 10.1038/sj.mp.4002124 PMC3952067

[26] Basu SN, Kollu R, Banerjee-Basu S (2009) AutDB: a gene reference resource for autism research. Nucleic Acids Res 37: D832–836. 19015121 10.1093/nar/gkn835 PMC2686502

[27] Allen NC, Bagade S, McQueen MB, Ioannidis JP, Kavvoura FK, et al (2008) Systematic meta-analyses and field synopsis of genetic association studies in schizophrenia: the SzGene database. Nat Genet 40: 827–834. 18583979 10.1038/ng.171

[28] Jia P, Sun J, Guo AY, Zhao Z (2010) SZGR: a comprehensive schizophrenia gene resource. Mol Psychiatry 15: 453–462. 20424623 10.1038/mp.2009.93 PMC2861797

[29] Zhang L, Chang S, Li Z, Zhang K, Du Y, et al (2011) ADHDgene: a genetic database for attention deficit hyperactivity disorder. Nucleic Acids Res 40: D1003–D1009. 22080511 10.1093/nar/gkr992 PMC3245028

[30] Konneker T, Barnes T, Furberg H, Losh M, Bulik CM, et al (2008) A searchable database of genetic evidence for psychiatric disorders. Am J Med Genet B Neuropsychiatr Genet 147B: 671–675. 18548508 10.1002/ajmg.b.30802 PMC2574546

[31] Yu W, Gwinn M, Clyne M, Yesupriya A, Khoury MJ (2008) A navigator for human genome epidemiology. Nat Genet 40: 124–125. 18227866 10.1038/ng0208-124

[32] Kao CF, Fang YS, Zhao Z, Kuo PH (2011) Prioritization and evaluation of depression candidate genes by combining multidimensional data resources. PLoS One 6: e18696. 21494644 10.1371/journal.pone.0018696 PMC3071871

[33] Cannon TD, Keller MC (2006) Endophenotypes in the genetic analyses of mental disorders. Annu Rev Clin Psychol 2: 267–290. 17716071 10.1146/annurev.clinpsy.2.022305.095232

[34] Gelder MG, Mayou R, Geddes J (1999) Psychiatry. New York: Oxford University Press.

[35] Gelder MG (1996) Oxford textbook of psychiatry. New York: Oxford University Press.

[36] Gelder M (2011) New Oxford textbook of psychiatry. New York, NY: Oxford University Press.

[37] American Psychiatric Association (2000) Diagnostic and Statistical Manual of Mental Disorders, Fourth Edition–Text Revision (DSM-IV-TR). Washington, DC: American Psychiatric Publishing, Inc.

[38] Gazzaniga MS, Ivry RB, Mangun GR (2009) Cognitive neuroscience : the biology of the mind. New York: W.W. Norton.

[39] Senior C, Russell T, Gazzaniga MS (2006) Methods in mind. Cambridge, MA: MIT Press.

[40] Allis CD, Jenuwein T, Reinberg D (2007) Epigenetics. Cold Spring Harbor, NY: Cold Spring Harbor Laboratory Press.

[41] Tollefsbol TO (2011) Handbook of Epigenetics: The New Molecular and Medical Genetics. Waltham, MA: Academic Press.

[42] Frodl T, Schule C, Schmitt G, Born C, Baghai T, et al (2007) Association of the brain-derived neurotrophic factor Val66Met polymorphism with reduced hippocampal volumes in major depression. Arch Gen Psychiatry 64: 410–416. 17404118 10.1001/archpsyc.64.4.410

[43] Seal RL, Gordon SM, Lush MJ, Wright MW, Bruford EA (2011) genenames.org: the HGNC resources in 2011. Nucleic Acids Res 39: D514–519. 20929869 10.1093/nar/gkq892 PMC3013772

[44] Magrane M, Consortium U (2011) UniProt Knowledgebase: a hub of integrated protein data. Database (Oxford) 2011: bar009. 21447597 10.1093/database/bar009 PMC3070428

[45] Barsky E, Giustini D (2007) Introducing Web 2.0: wikis for health librarians. Journal of the Canadian Health Libraries Association 28: 147–150.

[46] Scarabino T (2006) Atlas of morphology and functional anatomy of the brain. New York, NY: Springer.

[47] Handy TC (2005) Event-related potentials: A methods handbook. Cambridge, MA: The MIT Press.

[48] Sherry ST, Ward MH, Kholodov M, Baker J, Phan L, et al (2001) dbSNP: the NCBI database of genetic variation. Nucleic Acids Res 29: 308–311. 11125122 10.1093/nar/29.1.308 PMC29783

[49] Flicek P, Amode MR, Barrell D, Beal K, Brent S, et al (2011) Ensembl 2011. Nucleic Acids Res 39: D800–806. 21045057 10.1093/nar/gkq1064 PMC3013672

[50] Ashburner M, Ball CA, Blake JA, Botstein D, Butler H, et al (2000) Gene ontology: tool for the unification of biology. The Gene Ontology Consortium. Nat Genet 25: 25–29. 10802651 10.1038/75556 PMC3037419

[51] Kanehisa M, Goto S, Hattori M, Aoki-Kinoshita KF, Itoh M, et al (2006) From genomics to chemical genomics: new developments in KEGG. Nucleic Acids Res 34: D354–357. 16381885 10.1093/nar/gkj102 PMC1347464

[52] Vastrik I, D'Eustachio P, Schmidt E, Gopinath G, Croft D, et al (2007) Reactome: a knowledge base of biologic pathways and processes. Genome Biol 8: R39. 17367534 10.1186/gb-2007-8-3-r39 PMC1868929

[53] Huang da W, Sherman BT, Lempicki RA (2009) Systematic and integrative analysis of large gene lists using DAVID bioinformatics resources. Nat Protoc 4: 44–57. 19131956 10.1038/nprot.2008.211

[54] Huang da W, Sherman BT, Lempicki RA (2009) Bioinformatics enrichment tools: paths toward the comprehensive functional analysis of large gene lists. Nucleic Acids Res 37: 1–13. 19033363 10.1093/nar/gkn923 PMC2615629

[55] Keshava Prasad TS, Goel R, Kandasamy K, Keerthikumar S, Kumar S, et al (2009) Human Protein Reference Database–2009 update. Nucleic Acids Res 37: D767–772. 18988627 10.1093/nar/gkn892 PMC2686490

[56] Lopes CT, Franz M, Kazi F, Donaldson SL, Morris Q, et al (2010) Cytoscape Web: an interactive web-based network browser. Bioinformatics 26: 2347–2348. 20656902 10.1093/bioinformatics/btq430 PMC2935447

[57] Gottesman II, Shields J (1973) Genetic theorizing and schizophrenia. Br J Psychiatry 122: 15–30. 4683020 10.1192/bjp.122.1.15

[58] Gottesman II, Gould TD (2003) The endophenotype concept in psychiatry: etymology and strategic intentions. Am J Psychiatry 160: 636–645. 12668349 10.1176/appi.ajp.160.4.636

[59] Dai Q, Feng Z (2009) Deficient inhibition of return for emotional faces in depression. Prog Neuropsychopharmacol Biol Psychiatry 33: 921–932. 19394388 10.1016/j.pnpbp.2009.04.012

[60] Atkinson J, Colburn WA, DeGruttola VG, DeMets DL, Downing GJ, et al (2001) Biomarkers and surrogate endpoints: preferred definitions and conceptual framework. Clin Pharmacol Ther 69: 89–95. 11240971 10.1067/mcp.2001.113989

[61] Segman RH, Goltser-Dubner T, Weiner I, Canetti L, Galili-Weisstub E, et al. (2010) Blood mononuclear cell gene expression signature of postpartum depression. Mol Psychiatry 15: 93–100, 102. 10.1038/mp.2009.65 19581911

[62] Wong ML, Dong C, Maestre-Mesa J, Licinio J (2008) Polymorphisms in inflammation-related genes are associated with susceptibility to major depression and antidepressant response. Mol Psychiatry 13: 800–812. 18504423 10.1038/mp.2008.59 PMC2650233

[63] Spijker S, Van Zanten JS, De Jong S, Penninx BW, van Dyck R, et al (2010) Stimulated gene expression profiles as a blood marker of major depressive disorder. Biol Psychiatry 68: 179–186. 20471630 10.1016/j.biopsych.2010.03.017

[64] Pfleiderer B, Michael N, Erfurth A, Ohrmann P, Hohmann U, et al (2003) Effective electroconvulsive therapy reverses glutamate/glutamine deficit in the left anterior cingulum of unipolar depressed patients. Psychiatry Res 122: 185–192. 12694892 10.1016/s0925-4927(03)00003-9

