# Supplementary material for: MK4MDD: A Multi-Level Knowledge Base and Analysis Platform for Major Depressive Disorder
Source: PLoS One. 2012 Oct 5;7(10):e46335. doi: 10.1371/journal.pone.0046335 (PMC3465288; doi:10.1371/journal.pone.0046335)
Supplement: Supplementary File S1 — Criteria for keywords selection and all keywords employed to search MDD related publications in PubMed for MK4MDD. (DOC) [file pone.0046335.s001.doc]

**Supplementary file S1:** **Criteria for keywords selection and all keywords employed to search MDD related publications in PubMed for MK4MDD**

MK4MDD aimed to integrate different levels of biological components (gene, protein, cellular system/signaling pathway, neural system, cognition, and symptom [1]) that underlie major depressive disorder. A search formula **("major depression"[Title/Abstract] OR "MDD"[Title/Abstract] OR "unipolar depression"[Title/Abstract] OR "unipolar depressive disorder"[Title/Abstract] OR "major depressive disorder"[Title/Abstract]) AND ("XXX"[Title/Abstract] OR "XXX"[Title/Abstract] OR …OR"XXX"[Title/Abstract])** was designed to search and collect publications from PubMed. The five aliases of MDD were gotten from Wikipedia and Medical Subheadings (MeSH) terms of MDD. Keywords in different research levels (represented as XXX in the search formula) were selected according to the following criteria.

1. **Keywords targeting the first three research levels**. Keywords were decided by taking reference of several published databases on psychiatric disorders, such as ADHDgene [2], AutDB [3], SZGene [4] and SZGR [5]. Keywords for epigenetic studies were decided follow the representative textbooks [6,7].
2. **Keywords for neurobiological system**. This kind of keywords covers neurotransmitter systems, neuroendocrine and immune systems. The selection was based on several psychiatric textbooks [8,9,10] and important reviews about MDD [11,12]. Because neurobiological systems often contain multi-cell and multi-molecule, search results of this kind of keywords also partly overlap with the results by keywords in 1).
3. **Symptomatic keywords**. This kind of keywords covers the ‘symptoms’ and ‘cognition and behavior’ research levels, by following the diagnostic criteria for MDD in DSM-IV (diagnostic and statistical manual of mental disorders) [13]. It should be noted that the diagnosis about cognitive impairments of MDD in DSM-IV is described as ‘decreased ability to concentrate and think’, which is too general to describe the cognitive characteristics of MDD. So we set the data level ‘cognition and behavior’ to collect cognitive impairments and cognitive characteristics of MDD.
4. **Methodological keywords**. This kind of keywords corresponds to the data levels of ‘neural system’ and ‘cognition and behavior’. Major methods in neuroscience and cognitive science were included bead on several authoritative textbooks [14,15].

In conclusion, keywords were selected from different aspects by taking references of published database, paper and textbooks, so that they can cover all research levels on which MK4MDD is focusing. All keywords employed in PubMed search and their corresponding data levels are shown in Table S1

**Table S1** Keywords employed to search MDD related publications in PubMed for MK4MDD

| **Keywords category** | | **Specific keywords used in PubMed search** | **Covered data level** |
| --- | --- | --- | --- |
| Keywords targeting the first three research levels | ‘gene’, ‘epigenetic’, ‘protein’, ‘molecule’, ‘molecular’, ‘signaling’, ‘pathway’, ‘cell’, ‘cellular’, ‘neural’, ‘neuron’, ‘association’, ‘linkage’, ‘methylation’, ‘methylate’, ’histone’, ‘modify’, ‘modification’, ‘chromatin remodeling’ | | Genetic/epigenetic locus  Protein and other molecule  Cell and molecular pathway |
| Keywords for neurobiological system | ‘glutamate’, ‘γ-aminobutyric acid’, ‘monoamine’, ‘dopamine’, ‘norepinephrine’, ‘noradrenaline’, ‘epinephrine’, ‘5-HT’, ‘acetylcholine’, ‘nitric oxide’, ‘neurotransmitter’, ‘immune system’, ‘neuroendocrine’, ‘hypothalamic-pituitary-adrenal axis’, ‘HPA’, ‘hypothalamic-pituitary-thyroid axis’, ‘HPT’, ‘hypothalamic-pituitary-gonadal axis’, ‘hypothalamic-pituitary gland-growth hormone axis’, ‘HPGH’, | | Genetic/epigenetic locus  Protein and other molecule  Cell and molecular pathway  Neural system |
| Symptomatic keywords | ‘depressed mood’, ‘insomnia’, ‘hypersomnia’, ‘anhedonia’, ‘psychomotor retardation’, ‘low energy’, ‘fatigue’, ‘agitation’, ‘irritability’, ‘suicide intent’, ‘low self esteem’, ‘hopeless’, ‘worthlessness’, ‘guilt’, ‘decreased appetite’, ‘increased appetite’, ‘weight loss’, ‘weight gain’, ‘appetite’, ‘cognitive dysfunction’, ‘impaired memory’, ‘impaired concentrate’, ‘impaired executive function’ | | Cognition and behavior  Symptoms |
| Methodological keywords | ‘functional magnetic resonance’, ‘functional magnetic resonance imaging’, ‘fMRI’, ‘magnetic resonance imaging’, ‘magnetic resonance images’, ‘MRI’, ‘positron emission tomography’, ‘single photon emission tomography’, ‘PET’, ‘single photon emission computed tomography’, ‘SPECT’, ‘magnetic resonance spectroscopy’, ‘diffusion tensor imaging’, ‘electroencephalogram’, ‘EEG’, ‘event-related potential’, ‘event-related potentials’, ‘ERPs’, ‘ERP’, ‘magnetoencephalography’, ‘MEG’, ‘postmortem’, ‘eye-tarcking’, ‘eye movement’, ‘neuropsychological test’ | | Neural system  Cognition and behavior |

**References**

1. Cannon TD, Keller MC (2006) Endophenotypes in the genetic analyses of mental disorders. Annu Rev Clin Psychol 2: 267-290.

2. Zhang L, Chang S, Li Z, Zhang K, Du Y, et al. (2011) ADHDgene: a genetic database for attention deficit hyperactivity disorder. Nucleic Acids Res 40:D1003-D1009..

3. Basu SN, Kollu R, Banerjee-Basu S (2009) AutDB: a gene reference resource for autism research. Nucleic Acids Res 37: D832-836.

4. Allen NC, Bagade S, McQueen MB, Ioannidis JP, Kavvoura FK, et al. (2008) Systematic meta-analyses and field synopsis of genetic association studies in schizophrenia: the SzGene database. Nat Genet 40: 827-834.

5. Jia P, Sun J, Guo AY, Zhao Z (2010) SZGR: a comprehensive schizophrenia gene resource. Mol Psychiatry 15: 453-462.

6. Allis CD, Jenuwein T, Reinberg D (2007) Epigenetics. Cold Spring Harbor, N.Y.: Cold Spring Harbor Laboratory Press.

7. Tollefsbol TO (2011) Handbook of Epigenetics: The New Molecular and Medical Genetics.: Academic Press.

8. Gelder MG, Mayou R, Geddes J (1999) Psychiatry. Oxford ; New York: Oxford University Press.

9. Gelder MG (1996) Oxford textbook of psychiatry. Oxford ; New York: Oxford University Press.

10. Gelder M (2011) New Oxford textbook of psychiatry. New York, NY: Oxford University Press.

11. Krishnan V, Nestler EJ (2008) The molecular neurobiology of depression. Nature 455: 894-902.

12. Belmaker RH, Agam G (2008) Major depressive disorder. N Engl J Med 358: 55-68.

13. Association AP, DSM-IV. APATFo (2000) Diagnostic and statistical manual of mental disorders: DSM-IV-TR: American Psychiatric Publishing, Inc.

14. Gazzaniga MS, Ivry RB, Mangun GR (2009) Cognitive neuroscience : the biology of the mind. New York: W.W. Norton.

15. Senior C, Russell T, Gazzaniga MS (2006) Methods in mind. Cambridge, Mass.: MIT Press.
